# Reflections on a vulnerability framework for sustainability science

**DOI:** 10.4102/jamba.v15i1.1335

**Published:** 2023-07-26

**Authors:** B.L. Turner, Bing-Bing Zhou

**Affiliations:** 1 School of Geographical Science and Urban Planning, College of Liberal Arts and Sciences, Arizona State University, Tempe, United States of America; 2 School of Sustainability, College of Global Futures, Arizona State University, Tempe, United States of America; 3 School of International Affairs and Public Administration, Ocean University of China, Qingdao, China; 4 Key Laboratory of Coastal Science and Integrated Management, Ministry of Natural Resources, Qingdao, China

**Keywords:** sustainability science, vulnerability, new millennium, resilience, social-environment

## Abstract

The first vulnerability framework for sustainability science was published about two decades ago. It embedded vulnerability analysis into the foundational lens of sustainability and resilience research – the social-environmental system (SES) – and called for an integration of the vulnerabilities of the social and environmental subsystems as opposed to the dominating attention given at the time to societal vulnerability. The framework recognised that the environment itself is vulnerable to disturbances and that the interactions of the two subsystems create a system-wide vulnerability central to questions of sustainability or sustainable development. It also provided multiple components of analysis that should be considered if vulnerability research and assessments were to contribute more fully to sustainability themes. Using bibliometric analysis and attention to subsequent vulnerability publications, various impacts of this original framework on vulnerability studies were examined in the study, including its recognition by citations, citation pathways and fields of study, and the degree to which its various dimensions were employed. It was found that its large citation recognition was not necessarily matched by attention to the dimensions the framework proposed, noting several exceptions.

**Contribution:**

The authors interpreted this discrepancy to have followed from the analytical complexity fostered by the framework and to the significant proportion of vulnerability interests that was and remains focused on societal vulnerability as opposed to the social-environmental one, even in this moment in which sustainability in the Anthropocene has become a paramount query.

## Origins of a vulnerability framework for sustainability science (VASS)

**FIGURE 1:**
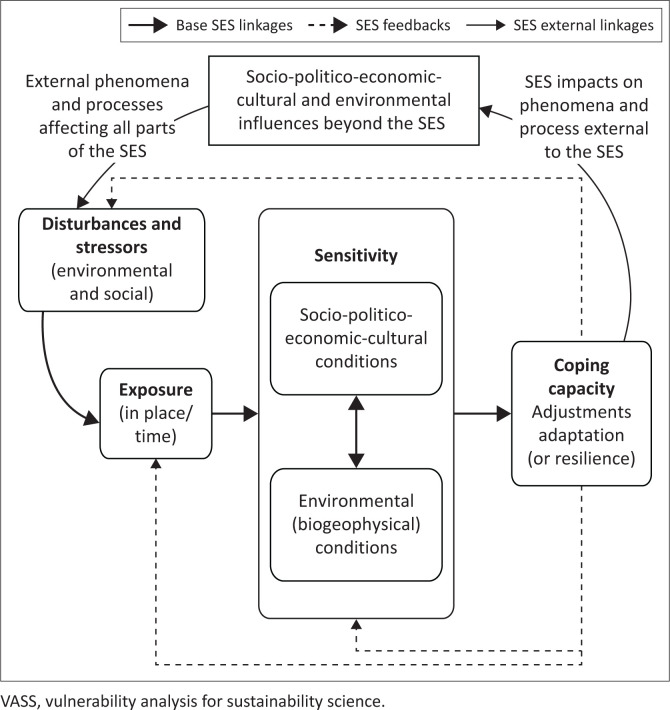
Simplified version of the VASS framework (Turner et al. 2003a).

The turn of the new millennium witnessed the formation of sustainability science (Kates et al. 2001; NRC 1999). This theme was a culmination of two decades of international, scientific attention to human-induced changes in the Earth system (e.g. International Geosphere-Biosphere Programme; Seitzinger et al. 2015), the issuance of the ‘Brundtland’ report by the World Commission on Environment and Development (WCED 1987) and the incipient recognition of the Anthropocene (Crutzen 2002). Sustainability science emerged as a new version of or direction in human–environmental science, in which the problem formation was anchored in the social-environmental system (SES; aka social-ecological system) (Clark 2007; Clark & Dickson 2003) and grounded in the interactions between the two subsystems. In this milieu, various frameworks linking the biophysical and societal dimensions of SESs emerged (Colding & Barthel 2019). One of these efforts merged concerns about disturbances on environmental systems with those of risk-hazard interests in social systems to create ‘a framework for vulnerability analysis for sustainability science’ (henceforth, VASS; Figure 1) applicable for SESs approaches (Turner et al. 2003a) and an accompanied illustration of its use (Turner et al. 2003b). The original framework did not employ the VASS acronym, and it was variously referred to by others as the vulnerability-sustainability or sustainability science or SUST framework (Fekete, Damm & Birkmann 2010; Popke, Curtis & Gamble 2016; Shen, Feng & Peng 2016), as well as the extended or expanded vulnerability framework (Armaş 2012; Preston, Yuen & Westaway 2011; Yu et al. 2020). The VASS label is warranted in this assessment, given the various vulnerability frameworks and models developed subsequently (e.g. Birkmann et al. 2013), some of which expand vulnerability dimensions beyond those in the VASS effort.

At its core, VASS advanced that SES vulnerability must treat both the social (inclusive of political, economic, and cultural dimensions) and environmental subsystems and their interactions. A full analysis would consider: (1) the multiple biophysical and social disturbances (also known as perturbations, hazards, shocks, stressors) to which the SES is exposed; (2) the sensitivity of the SES to that exposure, including its amplification or attenuation by the subsystem’s interactions; and (3) the coping (or adaptive) capacities of adjustments and adaptations of the two subsystems and their interactions. Feedbacks on disturbances, exposure and sensitivity occur throughout the VASS framework, and the outcomes have impacts on social and environmental phenomena and processes beyond the SES in question, which in turn affect the various dimensions of the VASS framework.

These characterisations of VASS have been recognised in subsequent vulnerability reviews and assessments (e.g. Preston et al. 2011). Less recognised, perhaps missed in much of the literature (but see Miller et al. 2010), was an equally important dimension of VASS – that the environmental subsystem itself maintains vulnerabilities that are not only important in their own right but which affect and are affected by SES dynamics. This dimension, of course, was a clarion call of the ecological sciences and resilience research examining SESs (Colding & Barthel 2019; Folke 2006). The original risk-hazard conceptual model from the social sciences did not account for this vulnerability. It also did not employ the term resilience – the persistence of SESs by way of their capacity to recover from a disturbance (Miller et al. 2010; Turner 2010) – but risk-hazard research at large addressed coping capacity. In the development of the VASS framework, the SES response dimension – coping capacity – was recognised as resilience (Figure 1).

The VASS framework (Turner et al. 2003a, 2003b) was developed not only to address specific SESs or problems associated with them but also with the conviction that systematic vulnerability analyses would ultimately identify common characteristics crosscutting similar SESs. Such generalities would not necessarily lead to the same outcomes, because the complexity of SESs generates variations in them. As such, the solutions to reduce vulnerabilities (i.e. increase resilience) must be malleable by case and treated through strategies of adaptive management. In addition, the VASS framework called for vulnerability analysis to account the restructuring of SESs after disturbances.

The VASS framework is only one version of subsequent models, framings, or other considerations of vulnerability and resilience (e.g. Cutter 1996, 2003; Hufschmidt 2011; Modica & Zoboli 2016; Smit et al. 1999). Other versions employ various terms and definitions, although the general meanings are similar (e.g. Adger 2000, 2006; Eakin & Luers 2006; Füssel 2007; Gallopín 2006). For the most part, vulnerability and resilience are viewed as the flip side of the same issue, focused on damages or recovery, respectively (Cutter et al. 2008; Nyerges et al. 2021; Turner 2010). Some research challenges the ‘flip side’ interpretation (e.g. Akter & Mallick 2013), however, owing to cases where the most damaged or sensitive units to a particular hazard are superior to the less damaged units in their recovery. The VASS and similar frameworks illustrate that such outcomes are a result of the adaptive capacity of the system in the recovery process, or to make the flip, their resiliency. For the most part, interpretations of the evidence indicate that increased impoverishment is associated with increased vulnerability and less resiliency to hazards of various kinds.

VASS also provided the linkages among the vulnerability dimensions, suggesting the potential for a middle-range explanatory construct to emerge (e.g. Roy Chowdhury & Turner 2019). Resilience does not have a framing similar to that of VASS but identifies the conditions of the SES – the amount (latitude), sensitivity (resistance) and proximity (precariousness) – for its change or not (Walker et al. 2004).

Pre-SESs vulnerability frameworks, foremost the risk-hazard (Burton et al. 1978) and pressure-and-release framings (PAR) (Blaikie et al. 1994; Wisner et al. 2004), employed linkages among dimensions as in the VASS framing, but these approaches focused on the vulnerability of people, not the SES as a whole. These two framings and that of resilience, however, informed the VASS effort. Importantly, VASS does not address the ‘root causes’ of SES vulnerability, as the PAR model does for social vulnerability (e.g. Wisner 2016). This omission followed in part because the addition of the environmental subsystem magnifies the complexity of vulnerability analysis and because the system formulation focuses attention on the integrative (i.e. feedbacks) dynamics at play more so than on the ‘root causes’.

Numerous framings of risk, hazards and vulnerability (with additional but related uses of other terms) have subsequently been developed, such as social vulnerability framings (e.g. Cutter 2003), for which several reviews exist (e.g. Birkmann et al. 2013; Preston et al. 2011). At the time of its publication, however, the VASS framework was the only one addressing vulnerability assessments explicitly through an SES lens, consistent with problem sets pervasive in sustainability research. Almost two decades have passed since that framing, one in which sustainability science has blossomed, including the amalgamation of international research programmes into the sustainability themes of Future Earth^1^, as has the SES lens or approach. In these circumstances, how has the VASS framework fared? This question is addressed through the clues of a bibliometric assessment and the content of literature that followed the VASS publication date of 2003.^2^ Interest in vulnerability research before that date may be found in various publications (e.g. Adger 2000; Eakin & Luers 2006; Füssel 2007; Moret 2014; Nyerges et al. 2021; Preston et al. 2011). Rather than address all the dimensions framed in the original VASS work, the authors focus on what they believe to be the most fundamental and the least subsequently entertained in the literature – vulnerability of the environmental subsystem and its interaction with the vulnerability of the SES as a whole.

## Data and methods

The authors searched on 01 June 2021 in Scopus for publications citing the VASS paper (Turner et al. 2003a), which led to 2277 papers. Those papers were further filtered by limiting the sample to those works published before 2021 in the English language as an article, book chapter, review, book or editorial in outlet sources of journals, books and book series. In total, the authors collected the bibliographic information of 1967 eligible papers for the bibliometric analyses. Specifically, the authors mapped thematic clusters based on topics mined from the titles and abstracts of the 1967 papers by using VOSviewer 1.6.16 (Van Eck & Waltman 2010) and mapped the research development paths based on citations among these papers with HistCite^TM^ (Garfield, Paris & Stock 2006). Examples of map interpretations may be found in Zhou, Wu and Anderies (2019). In addition, the authors used R (R Core Team 2013) to visualise the disciplinary landscape of the 1967 papers, as defined by their subject areas indexed by Scopus.

To investigate how key elements of VASS have been addressed in empirical vulnerability studies that cite VASS, a three-stage paper refinement of the 1967 papers noted above was conducted. These papers were limited to journal articles using the terms ‘vulnerability’ or ‘vulnerable’ in their titles, abstracts or keywords, creating a record of 1104 publications. These titles, abstracts or keywords were searched with the query of ([assess* OR evaluat* OR measur* OR quantif* OR indicator*] AND [data OR empirical OR evidence OR survey OR ‘case’ OR result*] AND [framework]), leading to 222 publications. Lastly, the second author screened the titles and abstracts to remove papers that contain those terms yet are not empirical or do not use the term framework in relation to vulnerability. In the end, 175 papers were determined as eligible for thematic coding.

Coding the 175 empirical papers involved consideration of 12 key features of VASS: (1) SES interaction; (2) feedbacks throughout the system; (3) vulnerability of the environmental or ecosystem subsystem; (4) multiple disturbances, both physical and social in kind; (5) attenuation or amplification of exposure or sensitivity due to subsystem interactions; (6) interactions of SESs or their phenomena and processes beyond the SES in question; (7) restructuring of SES after disturbance; (8) nonlinearity characteristics of the SES; (9) legacies in the system; (10) new vulnerability metrics or measures employed; (11) tests of the linkages in the system; and (12) approaches working backwards from unwanted outcomes to the disturbance. The second author read the full-texts of the papers to identify relevant information to determine the presence or not of the VASS-advanced features in the 175 papers. During the coding process, 28 papers were removed from the content analysis for irrelevance, leading to 147 coded papers. In addition, several papers included more than one case study, each of which was treated independently. As such, 157 cases in the 147 papers were examined regarding the VASS key features. In the process, feature 11 was revised from ‘test of linkages in the system’ to ‘the validation of the applied or newly developed vulnerability metrics or measures’ because almost none of the 157 cases explicitly tested the linkages in the system. Additionally, whether the studies were quantitative or qualitative-quantitative regarding the VASS-advanced dimensions was also coded. The first author coded 15 randomly selected papers out of the 147 papers (i.e. 10%) to cross-validate the coding reliability. The cross-validation by the first author produced an overall agreement of 96.16% and a Kappa Coefficient of 91.30%, indicating excellent inter-coder consistency.^3^ Subsequently, R was used to visualise the coding results of the content analysis.

### Ethical considerations

This article followed all ethical standards for research without direct contact with human or animal subjects.

## Results

### Subject areas and topical coverage of VASS-citing papers

**FIGURE 2:**
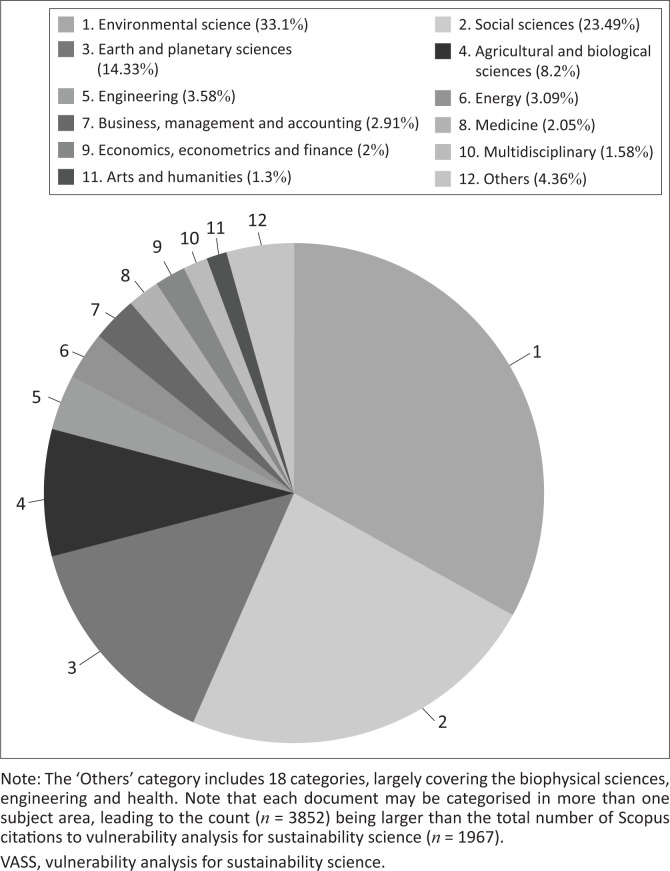
Citations to the VASS paper (i.e., Turner et al. 2003a) by Scopus subject area categories.

The VASS paper received 1967 citations in the Scopus database from peer-reviewed English outlets published before 2021. These articles were published in journals covering the full spectrum of the 27 major subject areas indexed in Scopus (Figure 2^4^). The highest-ranked journal subject areas citing VASS are environmental science (33.1%), social sciences (23.49%), Earth and planetary sciences (14.33%) and agricultural and biological sciences (8.2%), which account for about 80% of VASS citations. This subject range notwithstanding, only 1.58% of the VASS-citing papers were published in journals categorised by Scopus as multidisciplinary or those covering a large range of science fields and topics (e.g. *Nature, Science*).

**FIGURE 3:**
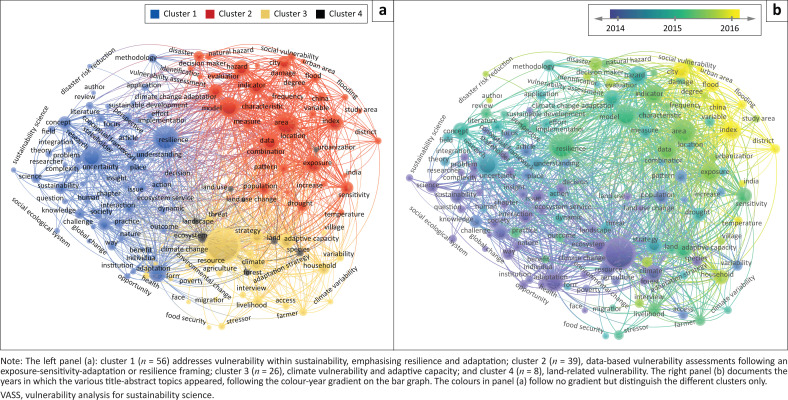
Thematic clustering and temporal evolution of the topics covered in the titles and abstracts of the 1967 VASS-citing papers.

Topics mined from the titles and abstracts of the 1967 VASS-citing papers add thematic details to the citation landscape of VASS. These details cluster into four main research themes (Figure 3a): vulnerability within sustainability, emphasising resilience and adaptation (cluster 1), covers 43.41% of the thematic topics; data-based vulnerability assessments following an exposure-sensitivity-adaptation or resilience framing (cluster 2), 30.23%; climate vulnerability and adaptive capacity (cluster 3), 20.16%; and land-related vulnerability (cluster 4), 6.20%. The temporal evolution of these topics (Figure 3b) suggests a research shift around the year 2015, with an orientation toward resilience, adaptive capacity, exposure, sensitivity and model construction (bottom left). Prior to 2015, the VASS-citing papers focused more on studies of climate change adaptation and sustainability. From 2016 onwards, the prevailing research topics relate to indicator-based assessments of social vulnerability, especially in contexts of urban flooding in China and India.^5^

### Literature development paths of VASS-citing papers

**FIGURE 4:**
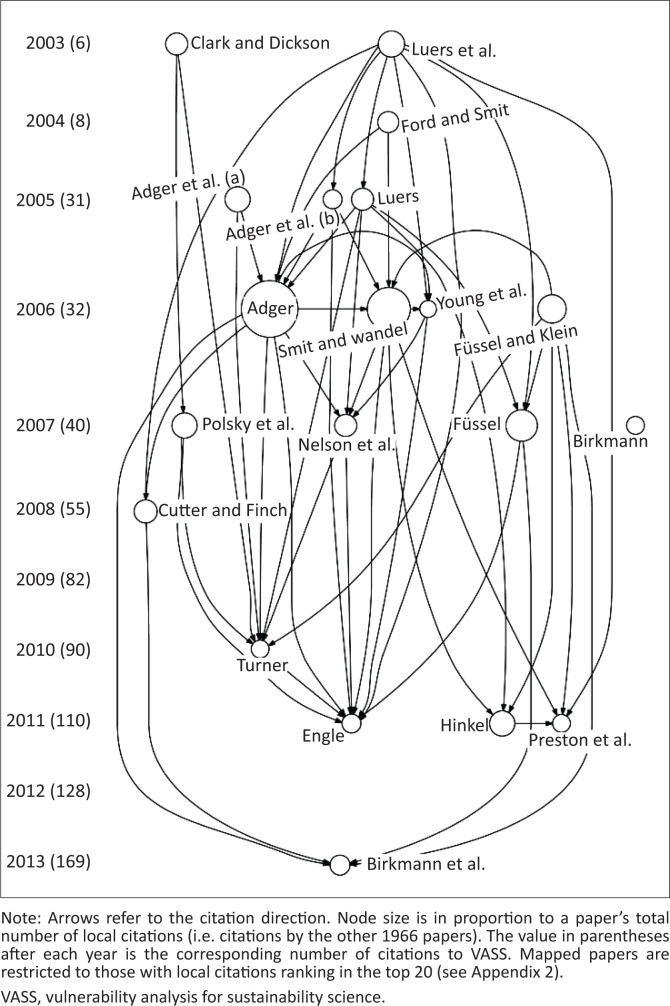
Literature development paths of the 1967 VASS-citing papers.

The topic mining is supported by historiographical analysis of the 1967 VASS-citing papers based on their citation linkages (Figure 4). Citation linkages among the top 20 papers that received the most local citations (i.e. citations to each sampled article by the other 1966 papers) show a cohesive research community. Full-text reading of these 20 seminal VASS-citing articles further reveals three research stages and current research frontiers of VASS-linked vulnerability research. The seminal papers prior to 2005 are mostly exploratory sectoral, regional or social operationalisations of the idea of SES vulnerability to climate change involving agriculture (Luers et al. 2003), community (Ford & Smit 2004) and coastal (Adger et al. 2005b) vulnerability. An exception is that by Clark and Dickson (2003) positioning SES vulnerability as advanced by VASS as a research frontier of sustainability science.

The VASS-proposed SES vulnerability concept was applied conceptually between 2005 and 2007. It was variously addressed regarding such topics as adaptation (Füssel 2007; Smit & Wandel 2006), often to climate change (Adger, Arnell & Tompkins 2005a; Füssel & Klein 2006), integrating risk, vulnerability, resilience and adaptation (Adger 2006; Birkmann 2007; Young et al. 2006), links to actor-centred vulnerability (Nelson, Adger & Brown 2007) and various analytical tools to address vulnerability (Luers 2005; Polsky, Neff & Yarnal 2007). During the 2008–2013 period, especially around 2011, several seminal papers were published providing critical reflections on vulnerability research, especially with regard to sustainability science. These involved advances in addressing social vulnerability (Cutter & Finch 2008), the distinctions and similarities between vulnerability and resilience (Turner 2010), treatments of adaptive capacity, vulnerability and measurement (Engle 2011; Hinkel 2011; Preston et al. 2011), and operationalisation of SES vulnerability through the MOVE (Methods for the Improvement of Vulnerability Assessment in Europe) framework (Birkmann et al. 2013).

### VASS features in VASS-citing studies

**FIGURE 5:**
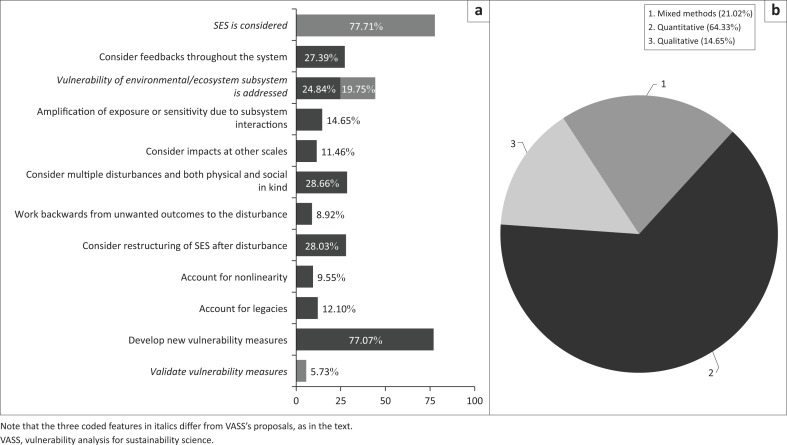
The proportion of 12 key features of VASS considered in the 157 coded cases of empirical vulnerability studies (a) and their approaches (b).

All of 1967 VASS-citing papers do not necessarily address SES vulnerability as proposed by VASS, an observation supported by this study’s content analysis of the 157 coded cases (above). In fact, the majority of the 12 key VASS features for operationalising SES vulnerability have received inadequate attention to date (Figure 5a). A significant majority of the cases considered both social and environmental components (77.71%), but not in terms of SES interactions as VASS proposed. Only around a quarter (27.30%) of the cases considered feedbacks throughout the SES system, typically feedbacks between parts of the system (e.g. coping capacity and sensitivity) or among the elements of either part. Although 44.59% of the cases addressed the vulnerability of the environmental or ecosystem subsystem, only 24.84% cases did so as part of the SES as a whole, whereas 19.75% addressed the environmental subsystem only. The paucity of attention to SES interactions and feedbacks is also reflected in the low fractions of cases addressing amplification of exposure or sensitivity as a result of subsystem interactions and impacts at multiple scales, which account for 14.65% and 11.46%, respectively.

A little over a quarter of these cases (28.66%) considered multiple disturbances, some addressing both physical (e.g. flooding, drought, sea level rise) and social (urbanisation, markets, Brexit) ones. A similar proportion of the cases (28.03%) considered the restructuring of SES after disturbances. Working backwards from unwanted outcomes to disturbance(s) and accounting for nonlinearities and legacies were only considered, respectively, in 8.92%, 9.55% and 12.10% of the cases.

Finally, over three-quarters (77.07%) of the cases were indicator-based assessments of regional, sectoral or social conditions that develop new vulnerability metrics or measures. Only 5.73%, however, attempted to validate or justify the reliability of such metrics, often by participatory judgement of indicator relevance or statistical alignment with participatory vulnerability ranking. In addition, very few of the coded studies elaborated cause-and-effect links, and almost none of them tested the links. Nonetheless, the dominance of indicator-based vulnerability assessments created a dominant quantitative approach (Figure 5b; 64.33%), followed by qualitative–quantitative mixed methods (21.02%). Only 14.65% of the cases are purely qualitative, providing vulnerability narratives, focus group results and ethnographic observations.

## Discussion

In the first decade of the new century, sustainability emerged as a major field of study, with SESs as either the phenomena or means of study. VASS was framed within this context, elevating it to the forefront of vulnerability discussions. This visibility is registered not only in the 1967 carefully identified citations in the Scopus database but also by the more than doubling of that number in Google Scholar, which includes more than articles alone. Either database indicates visibility ranging across a wide assortment of research fields (Figure 2). The 1967 papers identified (Figure 3) and the top 20 highly cited papers (Figure 4) point to a trajectory of attention common to new approaches applied to problems: exploration of the VASS-linked framework from 2003 to 2005; terminology clarification and conceptual and methodological advances from 2005 to 2007; and critical reflections and challenges from 2008 to 2013. The subsequent literature cannot be followed in the top 20 citation pathways because they have not yet had sufficient time to garner the requisite citation levels. The topics in the larger literature addressed, however, indicate that considerable attention beyond 2013 was given to indicator-based vulnerability assessments at the nexus of exposure, sensitivity and coping capacity (Figure 3b).

These three dimensions and the links among them appear as the most common VASS characteristics in the 157 cases of the 147 empirical vulnerability papers examined in detail in this study. Considering other articles, however, VASS-identified themes included: recognition of interactions with phenomena and processes beyond the immediate SES in question, feedbacks within and beyond the SES and attempts at fusing parts of resilience and vulnerability, especially in the context of sustainability problems (Birkmann et al. 2013; Cutter & Finch 2008; Hinkel 2011; Hufschmidt 2011; Nelson et al. 2007; Polsky et al. 2007; Preston et al. 2011; Prosperi et al. 2016; Wisner 2016).

It would be a mistake, however, to interpret the citation numbers as indicative of the acceptance of the VASS framework for vulnerability research, even within activities focused on sustainability topics. Recall that a large proportion of vulnerability studies are anchored in the interests of vulnerability of people or inhabited locations, consistent with origins residing in risk-hazard topics (e.g. Burton et al. 1978) and subsequently refocused on the causes of unsafe conditions confronting society (Blaikie et al. 1994). These interests remain dominant in the vulnerability literature compared to that of SES at large and especially to that of the environmental subsystem. Indeed, the array of vulnerability work associated with development and disaster interests, for example, as captured in the Sendai Framework for Disaster Risk Reduction (e.g. Aitsi-Selmi et al. 2015), emphasises social change more so than SES dynamics.

Few research efforts have attempted to link the two subsystems as VASS intended, likely because SES complexity proves problematic (Patt, Klein & De La Vega-Leinert 2005; Preston et al. 2011). Cutter (1996) presented a conceptual model of place vulnerability, the product of biophysical and social vulnerability, either of which interacted through geographic context and social fabric. This conceptualisation hinted of what could become an SES approach, although articulation of the interactions in question was not detailed. Notably, the complexity in question has increased as various dimensions of either subsystem have been specified and added as critical to a full vulnerability assessment (Birkmann et al. 2013; Wisner 2016). The authors are somewhat surprised, however, by the paucity of attention given to the vulnerability of the environmental (biophysical) subsystem and its interactions with other dimensions of the VASS framework, given the various ties of vulnerability, resilience and sustainability assessments.

As partners in the VASS development, Luers et al. (2003) and Luers (2005) undertook an ambitious effort to quantify the vulnerability to climate change of farmers within an irrigation scheme in the northern Mexico. This work connected environmental (e.g. soil and water) and social responses (e.g. cropping practices, yields) to provide a system-like vulnerability analysis, albeit considering only a few components in the system. Given the complexity of the SES approach, Luers et al. (2003) suggested that attention should be given to the vulnerability of the variables assessed rather than to their aggregation as a place or system at large. Luers (2005) expanded this study to create a three-dimensional surface tool to address SES vulnerability, one suggestive that the VASS approach may yield generalisable outcomes. To the authors’ knowledge, and to the loss of the SES vulnerability community, this tool has been underused in subsequent vulnerability studies.

Beyond Luers (2005), the authors’ examination of the literature uncovers only a few quantitative vulnerability studies in which the vulnerabilities of the environmental and social subsystems, or components therein, interact to address sensitivity and coping capacity, such as that undertaken by Wolff et al. (2015). While mixed methods and narratives of the VASS initiative attempted illustrate this orientation (Turner et al. 2003b), such methods have subsequently not been abundant either. Attention to environmental vulnerability exists from intellectual venues not linked to VASS (e.g. Jackson et al. 2004; Tran, O’Neill & Smith 2010) but tends not to address the social subsystem. Other vulnerability work treats variables in either subsystem but with outcomes focused on societal outcomes as opposed to the SES (Birkmann & Welle 2015; Cutter & Finch 2008; Farhan & Lim 2012; Nicholls et al. 2008; Raufirad et al. 2018). Not surprisingly, resilience research delves into the dynamics of the ecosystem (e.g. Chambers, Allen & Cushman 2019; Philippot, Griffiths Bryan & Langenheder 2021), with considerable attention to their interactions with the social subsystem (Cradock-Henry 2021; Siero et al. 2019), although the social subsystem may be treated more simplistically than the social science vulnerability community would desire (e.g. Côte & Nightingale 2011).

The initial approach of natural hazards research was variously challenged for its positivist (mainstream science) rationality as the base means of understanding knowledge (e.g. Ehrenfeld 1996) and its paucity of theory, foremost that explaining hazard vulnerability or the constraints of options to respond to hazards (e.g. Watts & Bohle 1993).^6^ The PAR model (e.g. Wisner et al. 2004) responded to such critiques, providing a means to address them empirically. In principle, the VASS framing can do so as well. To the authors’ knowledge, however, no efforts to test the causes of vulnerability, let alone those accounting for the SES as a whole, have been undertaken, consistent with the mainstream science approach to which VASS is associated.

Recognising the difficulties involved, multiple reviews and some frameworks have followed the SES lens or some of the dimensions for which the lens calls (e.g. Schröter, Polsky & Patt 2005), foremost the synergies between the components of the two subsystems relative to the SES vulnerability (Preston et al. 2011). The MOVE (methods for the improvement of vulnerability in Europe) framework comes close to the original VASS intent (Birkmann et al. 2013). Its sensitivity category (labelled susceptibility and fragility) recognises physical and ecological dimensions. The SES interacts with phenomena and processes operating at multiple spatiotemporal scales. Feedbacks with and beyond the SES are considered. The sensitivity and adaptation dimensions of the SES are associated with resilience. Explicit attention is given to the sensitivity of ecosystem functions and environmental services to disturbances, precisely that of the environmental subsystem called for in the VASS framework. Interestingly, however, attention to the adaptive capacity of the environmental subsystem is not explicitly mentioned.

The large majority of the features proposed in the VASS framework (Figure 5), however, such as accounting for nonlinear dynamics and system legacies or analysis that works from unwanted outcomes to the disturbance or hazard, appeared minimally in the literature examined in this study. In addition, the large proportion identified as considering the SES is achieved by enlarging the count to include environmental and social components in the assessments, not the vulnerability of the SES as an entity or the interactions of the two subsystems affecting each subsystem’s vulnerability. The one feature that has been undertaken with vigour is the development of new measures of vulnerability, such as those from energy (E_m_: embodied or consumed energy within a product or service) (Chang & Huang 2015) and land systems assessments (Wang et al. 2020) or from tailoring the conventional multidimensional indicator system to suit place-based vulnerability assessments (Wolff et al. 2015). This interest is not surprising given advances in data and analysis that permit quantitative assessments of different vulnerability, such as different hazards and coping capacity dimensions (i.e. adaptations).

## Conclusion and challenges

The VASS framework is clearly recognised among the array of research addressing vulnerability, especially so among such work linked to sustainability themes. It appears to resonate strongly in the need to consider the linkages of exposure-sensitivity-coping capacity and in the consideration of SES components. Other features proposed have drawn far less attention, save for the expected attention to vulnerability measures. Regardless of the many nods given to the SES framing, the overwhelming attention to research and assessments registering the vulnerability label, is focused on the social subsystem, foremost its sensitivity and coping or adaptive capacity–resilience (Adger 2006; Adger et al. 2013; Cutter 2003; Cutter, Boruff & Shirley 2003; Cutter & Finch 2008; Füssel 2007; Santhanam-Martin, Ayre & Nettle 2015). The dominant focus is consistent with the origins of risk, hazard and vulnerability analysis in the social sciences and the large application need from governmental organisations (e.g. Birkmann et al. 2013; Cutter et al. 2013; Preston et al. 2011).

This observation notwithstanding, a strong rationale exists for VASS-like dimensions to gain increasing attention. Sustainable development, in which the functioning of the Earth system to provide environmental services and measures and metrics that account for these services, such as inclusive wealth (Polasky et al. 2015), will continue to confront the research community and find its way to the interests of international science programmes (e.g. Intergovernmental Panel on Climate Change). The vulnerability–resilience nexus has been and will continue to be part of this interest, directed through the SES lens. Numerous challenges confront such efforts, three of which are identified here:

1. The literature indicates modest discrepancies or confusion among terminology, measures and metrics and other elements of sustainability work. Within the literature examined in this study, this observation applies to vulnerability, resilience, coping or adaptive capacities, and adaptation. Are vulnerability and resilience the flip sides of the same theme or not? Is coping capacity essentially resilience? How does adaptation differ from other adjustments to the changing conditions of the SES? Improved cohesion of the meanings among these and other terms among the different research communities should facilitate the integration of assessment communities.
2. While the authors applaud the increasing efforts to quantify vulnerability assessments, balanced by other approaches of course, far too little attention has been given to validation of the metrics and measures applied. Attention to internal (e.g. proposed cause–effect relationships) and external (e.g. stakeholder participation) validation is needed.
3. The VASS effort assumed middle-range theory building (Turner et al. 2020), which has been largely missing in the SES approaches to vulnerability. Given that such theory is lacking in the human–environmental sciences (i.e. testable, integrative explanation), a challenge for the VASS-linked vulnerability community is to identify and test design principles as has been advanced for common pool resources (Ostrom 1990).

## Footnotes

1 See https://futureearth.org/.

2 Other bibliometric assessments of vulnerability work exist, such as that by Fuller and Pincetl (2015) and Tschakert et al. (2013). None of these works, however, examine the citation links between VASS and subsequent vulnerability publications.

3 The small coding differences followed from distinctions made by the first author. Because almost none of the 147 articles examined interactions among the SESs (feature 1) and focused on SES vulnerability (feature 3), the first author coded features 1 and 3 as positive if they considered some component of either subsystem (feature 1), if the vulnerability of environmental subsystem is addressed in its own right and/or in addition to social vulnerability. For details of the coding and cross-validation, readers are referred to Online Appendix 1.

4 For a subject area classification scheme of journals, see https://libguides.eur.nl/courseresearchimpacts/scopus.

5 It is noteworthy that 2015 marked the emergence of the Sendai Framework for Disaster Risk Reduction, a United Nations effort to enlarge risk dimensions and link human health, development and climate change (Aitsi-Selmi et al. 2015). It is not clear how this framework affected the assessments of vulnerability and resilience addressed in the current study, a clarification that warrants further examination. References to this framework are not common in the literature examined in this study, perhaps because the Sendai Framework garners special attention from a community, many of whom publish in the *International Journal of Risk Disasters*, not necessarily aligned with the literature captured in this study’s bibliometric search.

6 The authors avoid elaborations of the critique of resilience and VASS as grounded in post-positivist or mainstream science. Engaging this critique would lead into the realm of explanatory perspectives (ontology, ideology and methods), and a vastly different topical theme, literature and vocabulary than that employed in this assessment. It is sufficient to note that structural and constructivist approaches can and have informed resilience and VASS research.
